# The Interplay Between Varicella-Zoster Virus and Giant Cell Arteritis: An In-Depth Narrative Review

**DOI:** 10.7759/cureus.93068

**Published:** 2025-09-23

**Authors:** Hasin Sharma, Madeline G Manuel, Alan Wang, Marc M Kesselman

**Affiliations:** 1 Osteopathic Medicine, Nova Southeastern University Dr. Kiran C. Patel College of Osteopathic Medicine, Davie, USA; 2 Rheumatology, Nova Southeastern University Dr. Kiran C. Patel College of Osteopathic Medicine, Davie, USA

**Keywords:** antiviral treatment, giant cell arteritis, glucocorticoids, infectious disease, rheumatology, varicella-zoster virus

## Abstract

Giant cell arteritis (GCA) is a large-vessel vasculitis that is commonly associated with inflammation of the blood vessels within the head and neck. The condition primarily affects older and immunocompromised individuals and is diagnosed through a combination of physical examination, laboratory tests, and, in some cases, biopsy. Most patients present with headaches, visual disturbances, and jaw claudication accompanied by low-grade fever, fatigue, and weight loss. Varicella-zoster virus (VZV) may play a role in the pathogenesis of GCA. VZV DNA has been identified within the cerebral arteries of a patient with fatal vasculitis of the central nervous system (CNS) without dermatologic presentation. Specifically, they identified VZV DNA within the cerebral arteries of a patient with fatal vasculitis of the CNS without dermatologic presentation, which has been further explored in more recent studies. While the presence of VZV within vascular endothelial cells and within temporal artery biopsies of patients with and without GCA suggests a possible overlap with GCA, debatable findings across recent studies, combined with inconsistent replication of biopsy techniques, have raised questions. The treatment of GCA involves high-dose corticosteroids, but in some cases, antivirals have been used when patients are resistant to corticosteroid treatment or if there is a strong clinical suspicion of VZV involvement. Multiple case reports have demonstrated the effectiveness of antivirals in treating GCA, but their validation remains limited by a lack of randomized controlled trials. In this narrative literature review, we explored current research efforts exploring the relationship between VZV and GCA. This study underscores the need for additional research to gain insight into the role of VZV in the pathophysiology of GCA and to establish evidence-based, more targeted treatment approaches that improve patient outcomes.

## Introduction and background

Donald Gilden's 1996 discovery

Despite advances in neurology and infectious disease, the origins of many vasculitic disorders of the central nervous system (CNS), namely giant cell arteritis (GCA), remain elusive. A pivotal discovery reshaped this landscape: in 1996, Gilden et al. reported a landmark case that first suggested a role for varicella-zoster virus (VZV) in GCA [1]. Specifically, Donald Gilden and his research team discussed a case of fatal waxing and waning CNS vasculitis that presented within a 73-year-old immunocompetent man without the presence of a typical zosteriform rash; he exhibited focal neurological deficits with multifocal infarcts of gray and white matter [1]. Although initial biopsies were inconclusive, subsequent analysis utilizing polymerase chain reaction (PCR) and immunocytochemistry techniques revealed VZV DNA and antigen localized within multiple large cerebral arteries [1]. The findings demonstrated that VZV vasculopathy can be highly focal, underscoring the necessity of sampling multiple arterial sites and employing diverse molecular techniques to accurately detect viral presence.

This discovery suggested that VZV may be associated with life-threatening vasculitis even without dermatologic manifestations. The findings suggest that early initiation of antiviral therapy may serve as a targeted, more effective therapeutic approach, in contrast to the immunosuppressive regimens traditionally used in managing vasculitis [1]. In addition, the investigators concluded that the detection of VZV via temporal artery (TA) biopsies should be considered in GCA-positive and/or GCA-negative patients who are nonresponsive to pharmacological treatment, given that VZV may exist as a subclinical phenomenon within patients diagnosed with GCA [1]. By uncovering the viral etiology of this case, the study aims to explore the clinical approach to CNS vasculitis and reinforce the importance of considering alternative infectious causes in similar clinical presentations.

Giant cell arteritis

GCA is a necrotizing granulomatous vasculitis that primarily affects medium- and large-sized blood vessels, most commonly involving the carotid artery [2]. Females, especially Caucasian females over 50 years old, most commonly present with GCA [3]. The incidence of GCA is approximately 20 cases per 100,000 individuals in adults over 50 years old [2]. In addition to genetic and autoimmune factors, such as polymyalgia rheumatica, infectious causes (i.e., VZV) have been shown to serve as possible triggers for GCA [2,3]. The pathogenesis of GCA is thought to involve the inappropriate activation of dendritic cells in medium and large vessels, leading to the recruitment and activation of CD4+ T-cells, and subsequently, the production of interleukin-6 (IL-6) and reactive oxygen species, which can result in vascular damage [2]. In some instances, interferon-gamma can stimulate the formation of giant cells, which release cytokines and vascular endothelial growth factor, contributing to vascular stenosis and occlusion [2].

GCA tends to present subacutely, but it occasionally may present abruptly with varying symptomatology [4]. Common symptoms include headaches, visual disturbances, jaw claudication accompanied by a low-grade fever, fatigue, and weight loss. Diagnosis is made through a combination of physical examination, laboratory tests, and, in some cases, biopsy [4]. Specifically, TA biopsies aid in the diagnosis of GCA [3,4]. The treatment of GCA commonly involves glucocorticoids; however, in some cases, antivirals have been used when patients are resistant to glucocorticoid treatment or if VZV involvement is considered in the diagnosis [1,4]. Furthermore, in cases where patients may experience adverse effects from glucocorticoids, it may be beneficial to add a biologic mediator, such as tocilizumab (TCZ) or methotrexate [4].

VZV

VZV is responsible for varicella (chickenpox) and HZV (shingles) [5,6]. It initially occurs as varicella in non-immune populations, then can establish latent infection as HZV [5]. Varicella has an incidence of approximately 7.2 cases per 1,000 individuals worldwide, while HZV has an average incidence of 8 cases per 1,000 individuals among patients over 65 years old [6,7].

It has been demonstrated that VZV induces infection by affecting multiple immune processes, including the expression of major histocompatibility complex class I and interferon response genes [6]. Following initial infection, the virus remains dormant in the dorsal and trigeminal ganglia until it is reactivated as HZV [6]. Individuals with HZV can present with fever, malaise, and vesicular lesions in a dermatomal distribution [5,6]. Diagnosis of HZV is clinical, but some testing, including Tzanck smear and detection of VZV antibody in the blood, may be helpful [7]. The most common pharmacological treatments for HZV are acyclovir or valacyclovir [7].

The course of VZV may be associated with multiple neurological complications, including meningoencephalitis, seizures, encephalitis cerebellitis, myelitis, postherpetic neuralgia, vasculopathy, and ocular disorders, which can result in paralysis, blindness, viremia, and death [8,9]. VZV-related vasculopathy may also contribute to neurological conditions such as ischemic and hemorrhagic strokes, cerebral aneurysms, arterial dissections, transient ischemic attacks, cerebral venous thrombosis, and spinal thrombosis [8].

While the etiology of GCA is multifaceted, evidence has evolved demonstrating the role of VZV in GCA pathogenesis. VZV is known to replicate not only in neuronal cells but also in endothelial cells of the arterial system and cerebral arterial cells [9,10]. When VZV infects cerebral vessels, it can lead to vasculopathy, and consequently, VZV-infected temporal arteries may contribute to the onset of GCA [9,10]. This can be explained by the fact that GCA commonly arises from an inflammatory process characterized by necrotizing granuloma formation within large and medium-sized arteries [8,9]. The presence of multinucleated giant cells, Cowdry A inclusion bodies, and/or epithelioid macrophages marks this inflammatory process [8,9].

VZV-induced GCA: diagnosis, treatment, and study aims

In the diagnosis of VZV-induced GCA, TA biopsy can be helpful, as approximately 73% of these biopsies tested positive for VZV DNA [4,11]. In cases of VZV-induced GCA, the TA biopsy typically reveals multinucleated giant cells and Cowdry A inclusion bodies [8]. In addition, nucleic acid amplification testing can further aid in detecting VZV DNA. One of the most serious complications of GCA is blindness, which can result from optic neuropathy or amaurosis fugax [11].

The elderly and immunocompromised populations are most vulnerable to developing VZV-induced GCA [12]. In addition, studies have indicated that some individuals may develop GCA following the administration of the live VZV vaccine, possibly due to a vaccine-induced cellular immune response affecting the arterial wall [12].

Treatment of GCA includes high-dose glucocorticoids; antiviral therapy may be added to treatment, though current evidence for its efficacy is limited to case reports rather than randomized controlled trials (RCTs) [9,11].

This review highlights the need for additional research to elucidate the role of VZV in the pathophysiology of GCA and to inform the development of evidence-based, targeted therapeutic strategies that aim to improve patient outcomes. Overall, the review has two aims. The primary objective is to examine the existing literature on the relationship between VZV and the pathogenesis of GCA, building on Gilden’s 1996 experiments. The second aim is to explore whether treatment for VZV-induced GCA includes high-dose glucocorticoids only or the inclusion of an antiviral.

## Review

Methodology

For this study, the following research question was asked: “To what extent should the potential association between VZV and GCA, as proposed in Gilden’s research, inform pharmacologic treatment strategies?”. Given the study's aims, a narrative review design was chosen to identify and generate a broad pool of relevant studies using various research methods (i.e., RCTs, review studies, and quantitative and qualitative research studies) to identify a consistent theme regarding the role of VZV in GCA pathogenesis. This study solely utilized a literature review. The authors did not utilize a patient selection process or use patient data.

**Figure 1:**
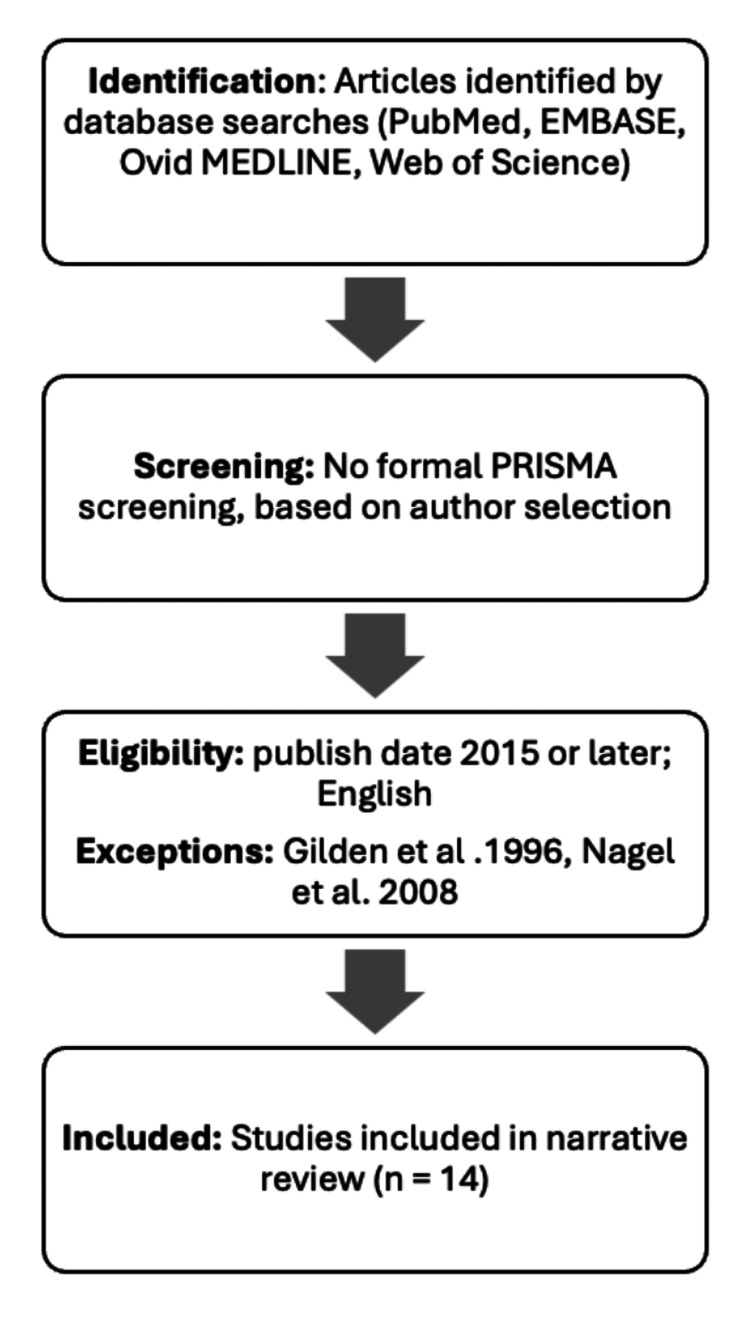
PRISMA-adapted flow diagram demonstrating study selection for narrative review PRISMA: Preferred Reporting Items for Systematic Reviews and Meta-Analyses

A literature search was performed during March 2025. The authors conducted independent searches of the articles in the study and performed screening based on an established inclusion/exclusion criterion. The search utilized the following databases: PubMed, Web of Science, EMBASE, and OVID Medline. The search terms “varicella zoster virus”, “giant cell arteritis”, “varicella zoster virus AND giant cell arteritis”, “giant cell arteritis AND treatment”, “varicella zoster virus AND treatment”, and “treatment of giant cell arteritis induced by varicella zoster virus” were used. The review included papers written in English or with a translation available, those published from 2015 or later, and those that addressed the chosen research question appropriately. The types of study designs included were systematic reviews, case reports, quantitative studies, and qualitative studies. This review excluded papers published before 2015, those that were not available in English, and/or did not align appropriately with the research question. Given that our paper focused on studies tied to Gilden et al. (1996), this paper was an exception to the exclusion criteria. The authors also decided to include Nagel et al. (2008) as an exception to the criteria, as the authors agreed that it contained valuable information for inclusion [1,9]. Fourteen studies were included for further analysis, as this paper is a narrative review; therefore, a formal Preferred Reporting Items for Systematic Reviews and Meta-Analyses (PRISMA) search strategy and screening were not performed. Instead, the authors were assigned sections of the paper to complete and selected literature to include in the paper. The selected literature was included if it addressed facets of the assigned section (i.e., if, in our discussion of the pharmacological treatment of VZV-induced GCA, the article addressed this topic while satisfying the inclusion criteria). Below is a modified PRISMA-style table (Figure 1) that outlines the identification and selection process for the chosen articles.

Results

Attached is a table that demonstrates the relevant studies used for the investigation. A total of 14 studies were included; the studies are labeled by reference order as they appear in the paper. These are demonstrated in Table 1 below. Another table in the Appendices section includes an additional list of references that contain supplemental evidence used in the creation of this review.

**Table 1:** Studies utilized for discussion and analysis in the investigation GCA: giant cell arteritis, VZV: varicella-zoster virus, MI: myocardial infarction, EBV: Epstein-Barr virus, ESR: erythrocyte sedimentation rate; CRP: C-reactive protein, 18F-FDG-PET: 18F-fluorodeoxyglucose positron emission tomography, MRI: magnetic resonance imaging, IL-1: interleukin-1, IV: intravenous, TA: temporal artery, CTLA4-Ig: cytotoxic T-lymphocyte-associated protein 4 immunoglobulin, IL-6: interleukin-6, TCZ: tocilizumab

Authors/publication date	Objective	Main findings
Gilden et al., 1996 [1]	Re-examine a case study from 1995 and investigate if VZV was an underlying etiology of the patient's vasculitis.	Upon re-investigation, they found VZV DNA and specific VZV antigen in the patient’s arterial samples. In cases of vasculitis where imaging reveals infarctions of both gray and white matter, investigating the etiology of VZV should be considered.
Lyons et al., 2020 [2]	Outline diagnostic methods, imaging modalities, and potential new therapies for GCA.	VZV is associated with vasculopathy, which can lead to granulomatous angiitis. This condition increases the likelihood of blood clot formation and may subsequently result in cardiovascular diseases such as ischemic stroke, MI, and heart failure. Some infections, including VZV, parvovirus B19, and EBV, may be associated with the development of GCA; however, the underlying mechanisms remain unclear. ESR and CRP levels can serve as initial diagnostic tools; however, imaging techniques such as cranial ultrasound, 18F-FDG-PET, and MRI are helpful for a more accurate diagnosis, as ESR and CRP are nonspecific indicators. TA biopsy is considered the gold standard for diagnosing GCA; it can also identify the presence of VZV. Regarding treatment, current guidelines recommend an initial glucocorticoid dosage of 40-60 mg daily.
Costanzo et al., 2023 [3]	Synthesize evidence from current studies in the literature on innovative treatments for GCA.	Systemic glucocorticoids, namely prednisone, are typically administered at a dosage of 40-60 mg daily until clinical symptoms improve. These are considered first-line therapy. Alternative treatment options include monoclonal treatments, such as TCZ, ustekinumab, and guselkumab. Additionally, Janus kinase inhibitors and IL-1 receptor antagonists may be possible future options for treating GCA.
Kennedy and Gershon, 2018 [8]	Discuss the clinical symptoms and complications of VZV, as well as preventive measures such as vaccination.	The common symptoms of VZV include pruritic skin lesions associated with chickenpox; there may be reactivation of VZV as shingles later in life. After the skin lesions heal, postherpetic neuralgia may occur. In immunocompromised individuals, complications such as encephalitis, vasculopathy, GCA, Guillain-Barré syndrome, and cranial neuropathy are frequently observed.
Gilden and Nagel, 2016 [9]	Explore if antivirals are beneficial in VZV-negative GCA patients. Review the role of VZV in GCA and propose a combination pharmacological treatment.	Currently, no trials have been conducted to determine whether antivirals and steroids provide additional benefits beyond steroids alone. However, antiviral treatment has successfully normalized symptoms and inflammatory markers. The appropriate dosage and duration of treatment have not yet been established. The presence of VZV in biopsies from both GCA-positive and GCA-negative patients reflects its potential role in triggering the immunopathology of GCA, suggesting the benefits of combined therapy with glucocorticoids and antiviral treatment with IV acyclovir.
Nagel et al., 2008 [10]	Provide a review of VZV vasculopathy cases, analyse imaging abnormalities, and examine the effects of treatment on patient outcomes.	VZV vasculopathy requires confirmation with virology and imaging studies. Patients demonstrated improved outcomes with treatment of acyclovir and steroids versus acyclovir monotherapy.
Ostrowski et al., 2019 [11]	Examine current literature to investigate a proposed link between VZV and the pathogenesis of GCA.	While some studies detect VZV DNA or antigen in TA biopsies of patients with GCA-like symptoms, the findings are inconsistent and have not been reliably replicated. VZV may mimic or trigger GCA in a subset of patients, but further controlled, reproducible studies are needed to confirm this.
Agger et al., 2020 [12]	Report a retrospective review using a cohort study to determine whether the introduction of live-attenuated VZV vaccine affected the incidence of GCA.	The live-attenuated VZV vaccine was found to be associated with an increased risk of GCA. This may be related to persistent arterial wall infection with VZV from live-attenuated vaccines, a cellular response, or a non-viral autoimmune response triggered by the vaccine.
Gilden and Nagel, 2015 [13]	Analyze TA biopsies to investigate a potential link between VZV and GCA.	VZV antigens were detected in all 4 GCA-positive TAs but not in normal TAs from the control group. The distribution of VZV antigen was predominantly located in the arterial adventitia and media.
Nagel et al., 2015 [14]	Assess the prevalence of VZV antigen in TA biopsies of patients with clinically suspected GCA who were histopathologically negative, and compare it to biopsy-positive and negative controls.	VZV antigen was detected in 64% of GCA-negative and 73% of GCA-positive biopsies compared to only 22% in controls. In some biopsy-negative cases, VZV was found alongside inflammation in the outer artery layer or tissue changes similar to GCA, suggesting that VZV may play a role in GCA pathogenesis or mimicry in biopsy-negative cases.
Freer and Pistello, 2018 [15]	Discuss the history, pathophysiology, epidemiology, and vaccinations for VZV.	Transmission of VZV occurs through inhalation of saliva droplets or direct contact with skin lesions. Prodromal symptoms include malaise, headache, and fever—these often precede a painful rash. Varicella infection was prevalent before the introduction of the vaccination.
Langford et al., 2017 [16]	To evaluate the efficacy and safety of abatacept, a CTLA4-Ig fusion protein, in preventing relapse of GCA compared to placebo.	Abatacept significantly prolonged remission compared to placebo (median 9.9 vs 3.9 months, p = 0.023), with no increase in adverse events. This supports its potential as a steroid-sparing agent in GCA treatment.
Stone et al., 2017 [17]	To assess if TCZ, an IL-6 receptor inhibitor, improves sustained remission in GCA patients undergoing glucocorticoid tapering.	TCZ, administered weekly or biweekly, in combination with a 26-week prednisone taper, resulted in significantly higher sustained glucocorticoid-free remission rates (56% and 53%) compared to placebo (14–18%), while also reducing total glucocorticoid exposure. Safety profiles were favorable, suggesting that TCZ may be a potential option for long-term management of GCA.
Costanzo and Ledda, 2024 [18]	To investigate current and emerging therapeutic options for inducing and maintaining GCA.	Glucocorticoids remain the standard treatment, but alternative therapies have been sought after due to the adverse effects of glucocorticoids. TCZ is the only biologic currently approved for GCA; however, emerging agents such as abatacept, baricitinib, and anakinra may become alternative treatment options in the future. Methotrexate and mycophenolate mofetil may also serve as effective adjuncts in reducing steroid exposure, although further trials are needed to confirm their long-term safety and efficacy.

Don Gilden's 1996 study and GCA pathogenesis

A Deeper Look: Gilden et al.’s (1996) Study

To investigate the cause of an unclassified fatal vasculitis affecting the CNS, Gilden and his team re-examined tissue samples from a 73-year-old immunocompetent man [1]. The patient had exhibited a progressive neurologic decline marked by mental status changes, focal neurological deficits, and multifocal infarcts in both gray and white matter. Still, notably, he never developed a zosteriform rash [1]. The researchers performed a detailed molecular and immunocytochemical analysis of formalin-fixed, paraffin-embedded sections of the basilar, vertebral, anterior, middle, and posterior cerebral arteries; histologic sections were stained using hematoxylin-eosin and van Gieson stains to evaluate inflammatory changes, disruption of the elastic lamina, and vessel pathology [1].

For viral detection, DNA was extracted from each arterial sample and subjected to PCR using primers specific for VZV, herpes simplex virus (HSV), and cytomegalovirus (CMV) [1]. Duplicate samples were tested to confirm reproducibility. Additionally, immunocytochemistry was performed on parallel tissue sections using rabbit antiserum against VZV open reading frame 63 protein, HSV-specific antiserum, and a mouse monoclonal antibody against CMV antigen [1]. Positive and negative controls, including virus-infected and uninfected cultured cells, were included to validate the assays.

By combining PCR and immunocytochemistry, the researchers aimed to increase the likelihood of detecting viral elements, recognizing that a patchy distribution could lead to false negatives if only one technique or a single vascular site were examined. Pathologic examination revealed a variable and patchy arteritis affecting the circle of Willis [1]. Some arterial segments were morphologically normal with intact internal elastic laminae, while others displayed intimal fibrosis, fragmentation of the elastic lamina, mononuclear cell infiltration, and, in some areas, fibrinoid necrosis [1]. Multinucleated giant cells were observed adjacent to disrupted elastic laminae, though no classic viral inclusion bodies were seen [1].

PCR analysis detected VZV DNA in two of the five cerebral arteries, the right posterior cerebral artery and the basilar artery. Still, no HSV or CMV DNA was identified in any of the vascular or brain samples [1]. Importantly, VZV DNA was not detected in brain tissue, further supporting a vessel-restricted infection [1]. Immunocytochemical analysis revealed the presence of VZV antigen in the right middle cerebral and posterior cerebral arteries, specifically within the thickened intima and occasionally in the media; no HSV or CMV antigens were detected in any sections [1]. These findings aligned with the PCR results and supported the diagnosis of VZV vasculopathy.

The distribution of inflammation and VZV antigen was highly focal, resembling "skip lesions," a feature commonly seen in GCA [1]. Some arteries and even some regions within the same artery were completely unaffected, emphasizing the critical need for extensive sampling and multiple detection methods [1]. Gilden et al. (1996) demonstrated that the detection of VZV DNA and antigen solely within arteries, but not in brain tissue, suggests that the virus primarily targets the vasculature in such cases rather than the brain parenchyma itself [1].

The patchy nature of the inflammation and viral presence underlined the importance of sampling multiple vascular sites and using complementary techniques such as PCR and immunocytochemistry [1]. If only a few sections or a single method had been used, this case might have been incorrectly classified as virus-negative, and the true cause of the vasculitis would have been missed.

Deeper Exploration of GCA Pathogenesis

Regarding research investigating the relationship between GCA and VZV, studies have been conducted since the early 2000s [11]. In 2014, Gilden and associates conducted a series of independent research studies and accompanying case reports on the potential association between GCA and VZV [13]. Due to the detection of VZV DNA or antigens in the TAs in GCA-negative and positive temporal arteries. Gilden et al. analyzed TA biopsies to determine if there was a causal link between VZV and GCA [13].

The experiments by Gilden and Nagel tested for an association between VZV and GCA. The researchers took four GCA-positive TAs and 13 control TAs and immunostained them with mouse monoclonal anti-VZV immunoglobulin-G1 antibodies [13]. If a section contained VZV antigen, the adjacent sections were subsequently stained [13]. In a separate experiment, Gilden found that VZV was positive in 74% of patients with GCA. The investigators identified 38% of them within the muscles adjacent to positive TAs, and 89% revealed GCA-positive histopathology in GCA-positive biopsies adjacent to those with VZV antigens, many with a skip pattern [11].

It was concluded that it is important to consider the detection of VZV in TA of GCA-positive and GCA-negative individuals because it could be a subclinical phenomenon, given that GCA is present among individuals over 50 years of age [2]. Gilden et al. initially identified this association through VZV’s pathogenesis, as the virus can infect the extracranial temporal, ophthalmic, and retinal arteries, which can cause symptoms similar to GCA [12]. Additionally, the investigators noted that the negative results for GCA and the absence of VZV presence in the TA do not rule out VZV infection, as anti-VZV immunoglobulin was present in the patient's cerebrospinal fluid. The association of VZV and GCA was also considered, given the presence of GCA pathology in skip areas of TA DNA adjacent to areas containing VZV antigen and DNA coding for VZV [12].

More recently, studies suggest that VZV may induce an immune-mediated effect, potentially leading to the co-occurrence of GCA and VZV [9,10,13]. One theory proposes that VZV may be present in GCA samples, albeit at levels undetectable by current tests, which could suggest that more TA biopsies may harbor VZV and GCA co-infections [10]. Given that GCA and VZV can be detected in skip lesions, this also presents a challenge in diagnosing GCA in VZV-infected TA. Nagel et al. (2016) suggested that GCA and multifocal VZV vasculopathy may have overlapping clinical and laboratory abnormalities, further supplementing Gilden’s research [9]. The researchers proposed that the absence of inflammation in those with VZV within the TA could be due to early treatment with glucocorticoids [9]. Additionally, Nigel et al. discovered that there may be a spectrum of GCA, which could be triggered by VZV infection, as the presence of VZV antigens is three times more likely in GCA-positive TA [13].

Meanwhile, other studies have demonstrated a lack of sufficient evidence to establish the role of VZV in the pathogenesis of GCA. This is due to the difficulty in diagnosing GCA and ensuring an accurate diagnosis, given that many diseases present with symptoms identical to those of GCA [12]. Additionally, there is a lack of substantial evidence indicating the presence of VZV in the trigeminal ganglion in cases of arteritis, regardless of whether it is GCA. Furthermore, studies by Nordberg et al. (2015) and Buckingham et al. (2018) have reported difficulties in replicating Gilden’s experiments and have not been able to confirm the presence of VZV antigen using PCR analysis of GCA-positive TA [12]. The researchers also failed to identify GCA arteries testing positive for VZV. Using antibody testing, the presence of a separate autoimmune disease caused by VZV cannot be ruled out, especially with the presence of positive IgG and IgM antibodies [12].

Researchers have highlighted inconsistencies in the methodology used to detect VZV [8,11,14]. Although Nagel et al. identified in a study on biopsy-positive and biopsy-negative samples that the presence of VZV may have a strong association with GCA, their findings have not been consistently verified by other research laboratories using PCR and immunohistochemistry techniques [14]. Sampling processing uniformity, tissue sectioning (including the exclusion of lesions), and the specificity of antibodies have been shown to interfere with reproducibility [11]. Additionally, the presence of viral DNA or antigens does not, in itself, diagnose pathogenicity. The VZV genome in most individuals, and particularly the aged, may remain latent without causing clinical disease [8]. The coexistence of VZV with vascular tissue at low viral titers suggests reactivation rather than the direct etiologic contribution towards GCA pathogenesis.

GCA treatment

Research on GCA treatment has expanded significantly in recent years. While glucocorticoids remain the first-line treatment approach, adverse effects associated with chronic use have identified an unmet need for steroid-sparing strategies or adjunctive treatment options [3].

Glucocorticoids are most commonly used to treat GCA due to their rapid anti-inflammatory effects and proven efficacy in preventing vision loss. Initial treatment typically involves oral prednisone, at doses of 40-60 mg/day, or intravenous (IV) methylprednisolone among patients with visual symptoms [2]. Symptoms, such as headache, fever, and polymyalgia rheumatica, generally improve within days of therapy initiation, while vascular complications may take longer to resolve [2].

Multiple adverse effects have been identified, which may result from taking these medications over the long term. These adverse effects may include osteoporosis, diabetes, hypertension, neuropsychiatric effects, and increased infection risk, with 50% to 100% of patients experiencing at least one significant adverse effect [17]. Relapses are also common during the tapering of glucocorticoid doses, with rates ranging from 43% to 79% [1]. Among elderly patients, long-term glucocorticoid use can be especially concerning, especially since this patient population is more vulnerable to these adverse effects [16]. For immunocompromised individuals, glucocorticoids further weaken the immune system, making the individuals more susceptible to opportunistic infections [8]. Given these risks, reduction of glucocorticoid exposure without jeopardizing disease control remains an unmet need and major therapeutic consideration.

Tocilizumab

Tocilizumab (TCZ) is a humanized monoclonal antibody that binds to both soluble and membrane-bound IL-6 receptors, thereby blocking IL-6-driven signaling pathways [2]. IL-6 is a key mediator in GCA immunopathogenesis, where it is involved in the differentiation of T-helper 17 cells (Th17), initiating hepatic acute-phase responses, such as CRP production, and driving vascular inflammation and remodeling [1]. TCZ has become the most effective biologic treatment for GCA and the only biologic agent approved by both the Food and Drug Administration and the European Medicines Agency for this condition [2].

The Giant-Cell Arteritis Actemra trial demonstrated significantly higher sustained remission rates and lower relapse rates among patients treated with TCZ plus a 26-week glucocorticoid taper compared to those on glucocorticoids alone [18]. TCZ use also demonstrated a noticeable reduction in cumulative steroid exposure by over 40%, indicating a decreased risk of glucocorticoid-related side effects [1]. Although generally well-tolerated, the adverse effects of TCZ use include elevated transaminases, neutropenia, dyslipidemia, and increased susceptibility to infections [18]. The drug also suppresses CRP and ESR, making clinical monitoring challenging in immunocompromised and older populations [1].

Abatacept

Abatacept is a fusion protein that inhibits co-stimulatory interaction between CD80/CD86 on antigen-presenting cells and CD28 on T cells. This results in a decrease in T-cell activation, a key mechanism of GCA pathogenesis [16]. In a randomized, double-blind, placebo-controlled phase II trial involving 49 patients with newly diagnosed or relapsing GCA, 48% of patients treated with abatacept remained in remission at 12 months, as compared with 31% in the placebo group [16]. Abatacept is associated with a relatively low risk of infection as compared with other biologics. While abatacept has demonstrated safety and moderate efficacy, the lack of larger-scale, phase III studies and regulatory approval has limited its use for this indication. As such, larger-scale clinical trials are needed to assess its role in clinical practice [16].

Targeting VZV

As noted above, an alternative hypothesis regarding GCA pathogenesis revolves around the possible involvement of VZV. GCA has historically been considered a primary autoimmune vasculitis of unknown etiology. Meanwhile, findings from several studies suggest that VZV might play a role in the pathogenesis of some patients. While no large-scale clinical trials have evaluated antiviral therapy among patients with GCA, several case reports and case series have suggested that patients with GCA-like symptoms, particularly biopsy-negative cases, may benefit from antiviral agents [9,11].

Gilden and Nagel (2016) published a case report of an 80-year-old man who presented with ischemic optic neuropathy (ION), with an associated history of ophthalmic-distribution zoster in the left eye [9]. He was diagnosed with GCA and given glucocorticoid treatment, which did not improve his symptoms. The researchers performed a TA biopsy and demonstrated the presence of the VZV antigen, and he was then treated with IV acyclovir. This resulted in recovery of vision, which supports the therapeutic potential of antiviral intervention [9].

Additionally, Ostrowski et al. (2019) provided a comprehensive review of clinical case reports evaluating the link between VZV and GCA, in which antiviral therapy was initiated when conventional glucocorticoid treatment alone was insufficient [11]. The researchers identified one case of a 54-year-old woman with diabetes mellitus who presented with ION and acute retinal necrosis. Although her TA biopsy was negative for GCA, amplifiable VZV DNA was detected in her vitreous fluid, and VZV antigen was present in the arterial adventitia [11]. Initial treatment with oral prednisone was ineffective, but after receiving intravitreal ganciclovir, high-dose oral acyclovir, and IV acyclovir, her condition improved significantly. The need to escalate from oral to IV antiviral treatment highlights the importance of early and aggressive antiviral management in cases with suspected viral involvement [11].

Another case highlighted by Ostrowski et al. (2019) involved a 72-year-old male who developed ophthalmic-distribution zoster, followed by VZV encephalitis and ION after initial treatment with glucocorticoids [11]. The patient’s TA biopsy was negative for GCA but positive for VZV antigen and DNA in skip lesions. This is significant because skip lesions, which are sections in the artery that exhibit patchy inflammation, can falsely indicate a negative TA biopsy result, posing a further challenge in diagnosis [11].

After antiviral treatment with oral valacyclovir, plus high-dose glucocorticoid treatment with IV methylprednisolone and oral prednisolone, which was withdrawn and glucocorticoids were tapered, the patient experienced a relapse of symptoms [11]. The patient showed improvement upon restarting IV acyclovir. This case highlights not only the diagnostic challenges of skip lesions but also the risks of immunosuppression in the absence of concomitant antiviral coverage in the context of VZV [11].

While glucocorticoids remain the primary therapy for GCA, cases of treatment failure, relapse, or progression to disseminated VZV vasculopathy, despite steroids, have been reported. This suggests that steroids alone may not be enough in patients with underlying VZV infection. Gilden and Nagel (2016) advocated for combining glucocorticoids with antiviral agents such as valacyclovir or IV acyclovir [9]. The investigators currently recommend prednisone (1 mg/kg) alongside valacyclovir (1 g three times daily), tapering steroids after four to six weeks, and continuing antivirals for an additional four to six weeks [9].

These case studies provide clinical context to the hypothesis that VZV may play a role in GCA pathogenesis. Consistent across case reports was the failure of glucocorticoids alone and the need for clinical resolution only after antiviral therapy was initiated. Additionally, the finding of VZV in biopsy-negative temporal arteries indicates that some patients with "seronegative" GCA may actually be experiencing VZV-induced vasculopathy, which can have therapeutic implications [11].

Meanwhile, an unmet need remains for larger-scale clinical trials to determine whether antivirals should be routinely administered to GCA patients, particularly those with negative biopsies but strong clinical suspicion, as well as to establish the ideal route, dose, and duration of antiviral treatment. Until such studies are conducted, these case reports can serve as a means of providing clinical evidence to support a more integrative approach to GCA treatment, especially among elderly and immunocompromised patients who are more susceptible to VZV reactivation.

Discussion

Overall, the current literature investigation reveals evidence in favor of a potential role of VZV in the pathogenesis of GCA. Multiple case studies discussed by Gilden et al. (2016) and Ostrowski et al. (2019) highlight clinical evidence in support of this relationship [9,11]. In terms of pharmacological treatment, case reports demonstrate that a combination of glucocorticoids and antivirals improves ophthalmological symptoms. Several treatments are being researched as potential alternatives to glucocorticoids, including abatacept and TCZ [15,16].

Although the investigation reveals consistencies with the current literature regarding a potential link between VZV and GCA, as well as treatment options, several limitations are noted in the existing literature. Despite significant advances in understanding and treating GCA, current research remains limited, particularly due to several critical gaps in the existing evidence, methodology, and generalizability. One limitation is that much of the evidence supporting antiviral therapy in GCA comes from anecdotal case reports, where symptom improvement was observed in patients treated with acyclovir or valacyclovir, often in conjunction with glucocorticoids, rather than from controlled clinical studies [9,11]. While compelling, these reports lack control groups and are susceptible to reporting bias, placebo effects, and confounding factors, such as concurrent immunosuppression, which is particularly prevalent among the elderly and immunocompromised individuals. These limitations underscore the need for rigorous evaluation of VZV-targeted strategies in GCA.

Since this review is a narrative study, a possible limitation is that it does not follow a formal PRISMA search or screening process. While this approach limits the ability to claim exhaustive coverage, the study design allowed the authors to capture a broad range of relevant literature, incorporating a variety of study designs, which illustrate key themes about the role of VZV in GCA pathogenesis and subsequent pharmacological treatment.

There have been no RCTs to date evaluating the efficacy of antiviral therapy (i.e., acyclovir, valacyclovir) in biopsy-positive or biopsy-negative GCA populations. Meanwhile, other pharmacologic agents such as TCZ have been tested in large-scale RCTs and shown to significantly reduce relapse rates and steroid exposure [15]. Similarly, abatacept has been shown to have only modest utility in sustaining remission among patients with GCA; however, it has not been widely adopted in clinical practice [16]. The lack of comparable high-level trials of antivirals warrants further investigation and caution for routine use in clinical practice.

An additional limitation is the diagnostic ambiguity, particularly with TA biopsy (the diagnostic gold standard for GCA), and the role of VZV in biopsy-negative GCAs. Skip DNA lesions, areas of inflammation alternating with normal tissue, can result in false-negative biopsies. In this context, the high detection rate of VZV in biopsy-negative patients raises the possibility that some cases labeled as GCA may instead represent VZV-induced vasculitis [13]. This theory is complicated by findings that VZV was also detected in up to 22% of normal, non-GCA controls. This suggests that VZV presence may not be sufficient to produce disease and that other immune or genetic cofactors might be involved [3, 9]. Without the ability to distinguish between causative and incidental viral presence, clinicians might overuse antivirals in indeterminate diagnostic cases.

A further limitation is that there is no current consensus regarding the optimal antiviral regimen for suspected VZV-associated GCA. Most reports involve IV acyclovir, while others use oral valacyclovir, but treatment durations vary widely [11]. Dosing strategies are extrapolated from herpes zoster or encephalitis protocols, with little evidence tailored to vascular inflammation. Additionally, combining antivirals with high-dose glucocorticoids introduces pharmacological interactions, complicating the interpretation of therapeutic outcomes [17]. Costanzo and Ledda (2024) highlighted the significance of early diagnosis and targeted immunomodulatory therapy among patients with GCA, but do not have recommendations for using antivirals, highlighting their uncertain role in clinical practice [18]. In the absence of clinical trials evaluating safety, efficacy, and cost-effectiveness, antiviral treatment remains an investigational adjunct rather than a standard component of care.

The hypothesized link between VZV and GCA also raises questions regarding vaccination as a preventive strategy. While the recombinant zoster vaccine is considered safe among immunocompetent individuals and even some immunocompromised individuals, its efficacy in preventing VZV-related vascular complications remains unproven [17]. Additionally, vaccination may not be feasible in patients with active vasculitis or those receiving immunosuppressive treatment [8].

Finally, epidemiologic and pathophysiologic gaps: population-based studies have not yet established a consistent association between herpes zoster incidence and GCA, even among high-risk populations such as the elderly and immunocompromised [11]. Despite the increased prevalence of VZV reactivation among these groups, attributable to either age-related decline in cell-mediated immunity or iatrogenic immunosuppression, epidemiological analyses have not demonstrated a resultant rise in GCA diagnosis [11]. Neither seasonal clustering nor geographic trends have shown clear correlations between regional herpes zoster outbreaks and GCA surges [11]. Additionally, the mechanisms by which VZV might trigger granulomatous vasculitis in immune-privileged arteries remain largely theoretical [9]. While VZV can spread transaxonally and has been associated with other forms of vasculopathy, clear mechanistic links to GCA have yet to be established [14]. Overall, these gaps suggest that the VZV-GCA association remains uncertain and warrants further investigation into its mechanistic and epidemiological aspects.

## Conclusions

The findings by Gilden et al. (1996) fundamentally reshaped the clinical understanding of vasculitis by identifying VZV as a potential underlying mechanism of large-vessel inflammation, even in the absence of classical symptoms. Although subsequent research has provided additional support for the involvement of VZV in GCA, limited large-scale studies, conflicting data, and methodological challenges have continued to fuel the debate. Meanwhile, the growing recognition of vascular tropism associated with VZV underscores the need to consider alternative diagnostic and therapeutic strategies, particularly among older and immunocompromised populations/or those unresponsive to first-line glucocorticoid treatment and/or oral antiviral therapy. This review suggests that future research should focus on refining detection methods, validating antiviral therapies, and redefining GCA management to ensure that infectious contributors, such as VZV, are neither overlooked nor undertreated.

## Appendices

**Table 2:** Studies included to support the context of the introduction VZV: varicella-zoster virus, MI: myocardial infarction, CVD: cerebrovascular disease, HZV: herpes-zoster virus, GCA: giant cell arteritis

Study	Objective	Findings
Tofade and Chwalisz, 2023 [19]	Describe recent neuro-ophthalmic manifestations of VZV in the literature.	VZV can lead to serious complications such as acute retinal necrosis, optic neuropathy, orbitopathy, Ramsay Hunt Syndrome, meningoencephalitis, and vasculitis. Therefore, prompt diagnosis and timely management are essential.
Cersosimo et al., 2022 [20]	Examine the relationship between VZV and cardiovascular disease.	VZV is associated with vasculopathy, which leads to granulomatous angiitis. This condition increases the risk of blood clot formation and, consequently, cardiovascular diseases like ischemic stroke, MI, and heart failure. Patients with CVD are more likely to experience severe complications from VZV infections.
Patil et al., 2022 [21]	Discuss current updates related to clinical presentations, complications, and management of HZV.	Clinical symptoms in pre-eruptive, acute, and chronic stages can present differently. Known complications are herpes zoster ophthalmicus, postherpetic neuralgia, meningitis retention syndrome, acute colonic pseudo-obstruction, erythema multiforme, and vasculitis.
Harbecke et al., 2021 [22]	Discuss various forms of vaccines that help prevent HZV infections.	Live-attenuated VZV vaccine and adjuvanted VZV glycoprotein E subunit vaccine are effective against VZV and safe to use in selected immunocompromised persons. Wider distribution of VZV vaccines will enhance public health.
Gershon et al., 2015 [23]	Discuss the pathophysiology of VZV infections, symptoms, complications, and vaccination as a primary preventive measure.	The incidence of zoster is highest among preschool-aged children in temperate climate areas. Vaccinations are routinely recommended during childhood. The average age for zoster infection, which is the reactivation of the VZV, is 50 years. Neurological complications associated with VZV can include paresis, neuralgia, meningoencephalitis, vasculopathy, GCA, and ophthalmic complications.
